# CT-Guided Online Adaptive Hypofractionated Radiotherapy for Primary Bladder Cancer: A Report of Two Cases

**DOI:** 10.7759/cureus.67318

**Published:** 2024-08-20

**Authors:** Ryan Davis, Colton Ladbury, Kevin Tsai, Borna Maraghechi, Chengyu Shi, Rose Li, Scott Glaser, Savita Dandapani, Jeffrey Wong, Terence Williams, Percy Lee

**Affiliations:** 1 Radiation Oncology, City of Hope National Medical Center, Duarte, USA; 2 Radiation Oncology, City of Hope Orange County Lennar Foundation Cancer Center, Irvine, USA

**Keywords:** hypofractionated rt, ct-guided adaptive radiotherapy, bladder cancer, adaptive radiation therapy, varian ethos

## Abstract

Trimodality treatment for bladder cancer, consisting of maximal transurethral resection of the tumor followed by concurrent chemoradiotherapy, is an attractive management option with curative and organ-sparing intent. However, such treatment can be associated with acute toxicities related to the large treatment margins required due to daily variation in bladder filling, with resultant bladder, bowel, and rectal toxicity. Adaptive radiation, which accounts for inter-fraction variations in bladder size, allows the confident delivery of radiation to bladder cancer with smaller margins, with the potential to reduce toxicities without the associated risk of compromising the target coverage. Herein, we present a case series of two patients with primary bladder cancer who were treated with computed tomography (CT)-based online adaptive hypofractionated radiotherapy using the Ethos system (Varian Medical Systems, Palo Alto, CA, USA). The first is an 83-year-old male with a remote history of prostate cancer treated with radiotherapy, who received adaptive radiotherapy as a means of decreasing the required margin size and optimizing planning based on adjacent bowel to reduce the risk of re-irradiation. The second patient is a 78-year-old male with node-positive bladder cancer, which necessitated whole pelvis radiotherapy, who underwent adaptive treatment (25 fractions) as a means of sparing cumulative dose to the bowel while ensuring suitable target coverage. In both cases, the clinical target volume consisted of the entire bladder (± nodes) with a planning target volume expansion of 7 mm. During treatment, daily cone-beam CT scans were acquired and used to generate adapted plans. These plans were compared to the original plans, with attention to target coverage and dose to organs at risk. For all 45 fractions, the adaptive plan was selected, primarily as a means of improving target coverage. This case series demonstrates that the adaptive Ethos system effectively delivers treatment for primary bladder cancer. Further data are needed for clinical toxicity outcomes and the efficacy of this approach.

## Introduction

The management of muscle-invasive bladder cancer (MIBC) includes neoadjuvant cisplatin-based combination chemotherapy followed by radical cystectomy as the mainstay treatment approach [1-4]. However, radiation therapy has been found to be a compelling treatment alternative for bladder cancer.

Radiation serves as a viable alternative for patients desiring an organ-sparing approach, who are poor surgical candidates due to age or comorbidities, or for personal preference [1,5,6]. In patients with MIBC who opt for treatment with radiation, a trimodality treatment (TMT) approach is used with concurrent chemoradiotherapy and maximal transurethral resection of bladder tumor (TURBT) [1]. In favorable patients, concurrent chemoradiotherapy consists of cisplatin or mitomycin with 5-fluorouracil (5-FU) and radiation to 60-66 Gy in 2 Gy per fraction [7]. However, data suggest that a hypofractionated approach to 55 Gy in 2.75 Gy per fraction may be equally or more effective [8].

Of the patients with intact bladders who complete TMT, 75% will have normal bladder function. This comes at the cost of ~20% of patients developing persistent bowel symptoms [9]. Despite this, quality-of-life metrics favor TMT over radical cystectomy [10]. Nevertheless, the large treatment volumes necessary to account for day-to-day anatomical variation of the bladder are directly responsible for the acute and chronic bowel toxicity associated with treatment [11].

Adaptive radiotherapy is an emergent technology that overcomes many limitations of 3D conformal treatment. By utilizing daily computed tomography (CT) imaging and artificial intelligence (AI), tighter margins can ensure coverage of the target while reducing the dose to normal tissues [12,13]. Efforts are currently underway to investigate outcomes compared to standard conventional treatment [14,15]. In this report, we describe our experience in treating two patients with MIBC using an adaptive radiation approach.

## Case presentation

Patient 1 (pelvic re-irradiation)

**Figure 1:**
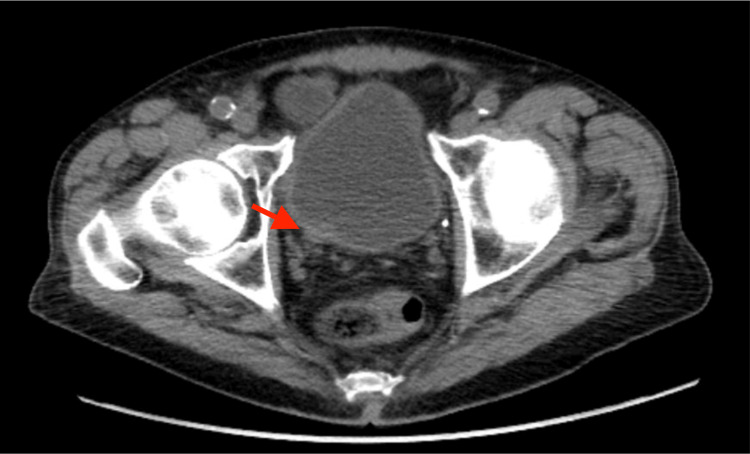
Image from CT pelvis depicting lesion at the ureteral insertion of a bladder CT: computed tomography

An 83-year-old male with a previous medical history significant for prostate cancer, treated with definitive external beam radiation in the 1980s, completed a urinalysis due to concern for a urinary tract infection (UTI). He was found to have evidence of infection with associated microscopic hematuria. The patient’s microscopic hematuria did not resolve with antibiotics on subsequent examinations. The patient was subsequently evaluated by urology. Cystoscopy revealed a bladder mass with concern for involvement of the ureteral orifice. A CT urogram was obtained and was notable for a 1.1 x 0.9 cm lesion at the ureteral insertion of the bladder (Figure 1).

The patient subsequently underwent TURBT and was noted to have growths located along the left and right walls, extending down and around the right ureteral orifice. The largest of these measured up to ~6 cm in diameter. The growths were finely papillary and associated with significant surrounding mucosal erythema, and were highly suspicious for urothelial carcinoma. These were excised using bipolar loop cautery and sent to pathology for examination. The tumor around the right ureteral orifice was resected and sent as a separate specimen, as it appeared to be deeply invasive and encased the right distal ureter. The end of the right ureter was deeply resected. Pathology of the right ureteral specimen returned as high-grade urothelial carcinoma demonstrating invasion into the muscularis propria. The remaining specimens demonstrated reactive changes without evidence of malignancy. A staging fluorodeoxyglucose (FDG)-positron emission tomography (PET)/CT was obtained, without evidence of nodal or distant disease.

The patient met with urology and was offered a cystoprostatectomy. However, he was hesitant to undergo surgery and sought additional treatment options. He was seen by an outside radiation oncologist, who felt that re-irradiation in the pelvis for his bladder cancer, after the remote history of prostate cancer irradiation, was too high-risk. The patient presented for a second opinion. We did not have access to the patient’s prior treatment records from the 1980s, although presumably, a significant dose was delivered to the small bowel, sigmoid colon, and rectum. However, given the long interval since his prior radiation, it was determined that he would be an appropriate candidate for radiation using CT-guided online adaptive radiotherapy to shrink the planning target volume (PTV) margin in the context of pelvic re-irradiation.

When treating patients with limited-stage bladder cancer with an organ-sparing curative intent, patients are frequently treated according to the BC2001 protocol [16]. In this protocol, patients undergo CT simulation in the supine position with an empty bladder. The entire bladder is contoured, and an isometric PTV expansion of 1.5-2 cm is used to account for day-to-day variation in bladder volume. This expansion can include a small bowel, as the bowel contents can shift in tandem with bladder filling. If the patient has bladder filling greater than at the time of simulation, a portion of the bladder can expand outside of the anticipated volume, and under-coverage of the target may occur. Conversely, if the bladder is underfilled compared to the time of simulation, a greater proportion of small bowel could enter the PTV volume and receive a prescription radiation dose.

We created a plan according to BC2001 based on images acquired at the time of CT simulation. The patient underwent simulation in the supine position with an empty bladder protocol. The treating MD was responsible for the clinical target volume (CTV) and PTV targets. The therapist contoured the organs at risk (OARs), which were later adjusted by the MD. The entire bladder CTV was contoured, and a 1.5 cm PTV expansion was made. OARs, including the femoral heads, rectum, and small bowel, were delineated. Constraints were those used per BC2001 protocol. The small bowel at the time of CT simulation appeared to abut the dome of the bladder. As a result, when optimizing for PTV coverage, the volume of small bowel receiving 42 Gy or greater was ~60 cc (V42Gy = 60 cc). This was substantial enough to confer an unacceptable risk - more so in the setting of re-irradiation.

**Figure 2:**
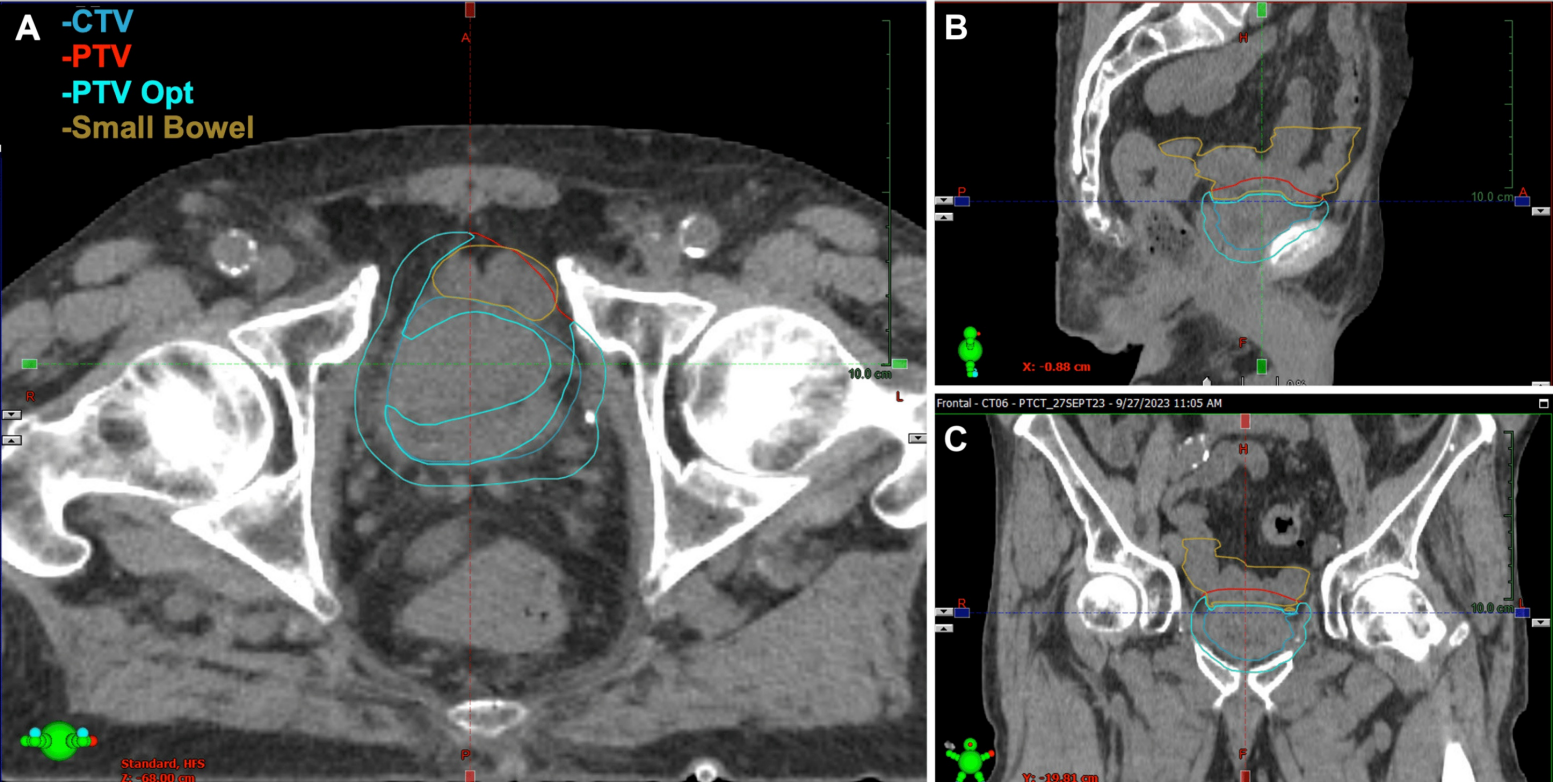
Contours for Patient 1 Axial (A), sagittal (B), and coronal (C) depictions of relevant contours CTV: clinical target volume; PTV: planning target volume; Opt: optimized

When using adaptive intensity-modulated radiotherapy (IMRT), tighter PTV margins are acceptable, as daily cone-beam CT (CBCT) imaging and plan adaptation account for the observed anatomical changes. Therefore, the patient was planned for daily CBCT adaptive IMRT with the Ethos system (Varian Medical Systems, Palo Alto, CA, USA), using an isometric PTV margin of 7 mm (Figure 2), which was used to create an initial baseline plan - termed “scheduled plan.” The patient was planned to receive a total dose of 55 Gy in 20 fractions. During adaptation, target volumes and OARs were initially contoured by the built-in AI models. For the initial fraction, the treating physician contoured the bladder and relevant OARs. For subsequent fractions, therapists and physicists completed preliminary contours, which were adjusted and/or verified by the physician. In total, the contouring phase took between 10 and 15 minutes. Treatment plans were generated prioritizing dose reduction to OARs. As such, daily plans were optimized based on separate PTV structures cropped 5 mm from the small bowel to allow further dose reduction to the small bowel. The dosimetric OAR goals for the rectum and femoral heads were achieved across all plans and, thus, were not included in the optimization.

**Figure 3:**
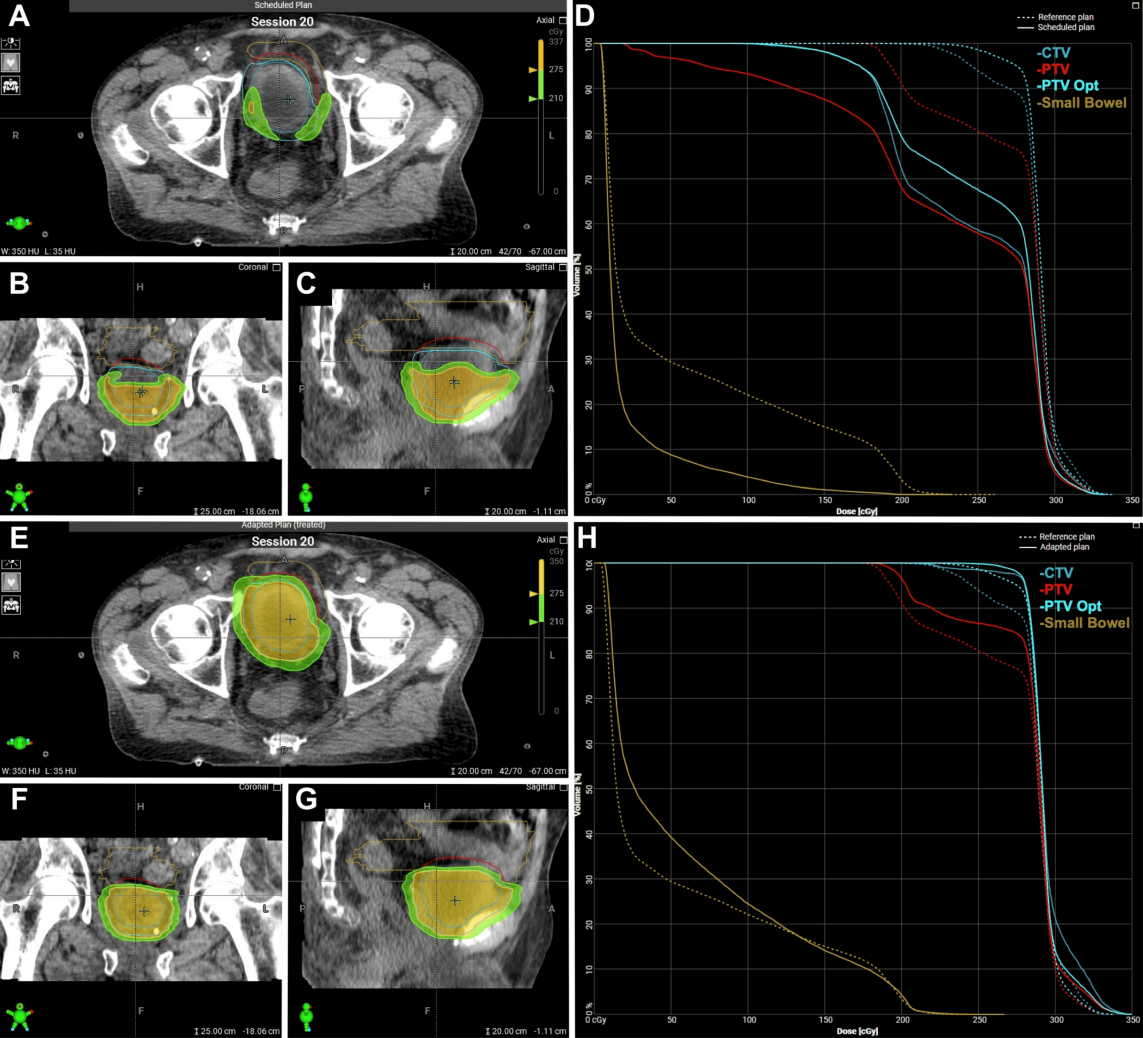
Scheduled and adapted radiation treatment plans for Patient 1 Axial (A), sagittal (B), and coronal (C) depictions of scheduled plan. Dose-volume histogram (D) of scheduled plan. Axial (E), sagittal (F), and coronal (G) depictions of the scheduled plan. Dose-volume histogram (H) of the adapted plan. CTV: clinical target volume; PTV: planning target volume; Opt: optimized

The patient completed his course without significant toxicity. He reported mild Common Terminology Criteria for Adverse Events (CTCAE) grade 1 urinary urgency and nausea towards the end of treatment. Due to a combination of target coverage and dose to the small bowel, the adapted plan was selected for all 20 fractions. The median patient time on the table was 35 minutes (range, 28-66). Examples of the initial plan and adapted plan for a given fraction's anatomy are shown in Figure 3.

**Figure 4:**
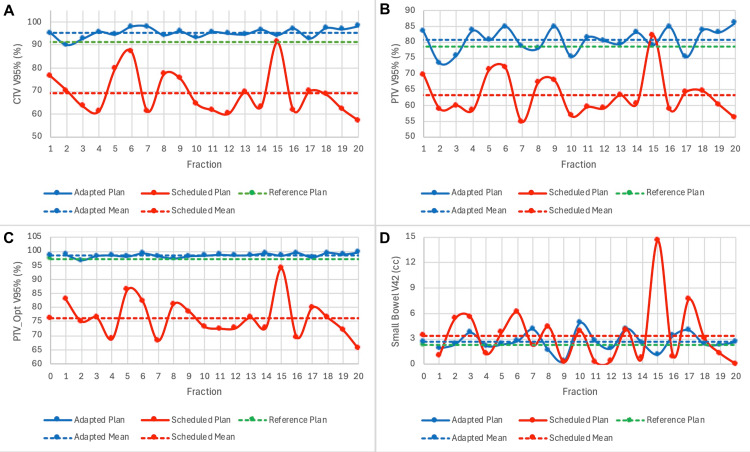
Daily dosimetry for target volumes and organs at risk for Patient 1 Daily CTV V95% (A), PTV V95% (B), PTV_Opt V95% (C), and Small Bowel V42 are plotted over the course of treatment CTV: clinical target volume; PTV: planning target volume; Opt: optimized

**Figure 5:**
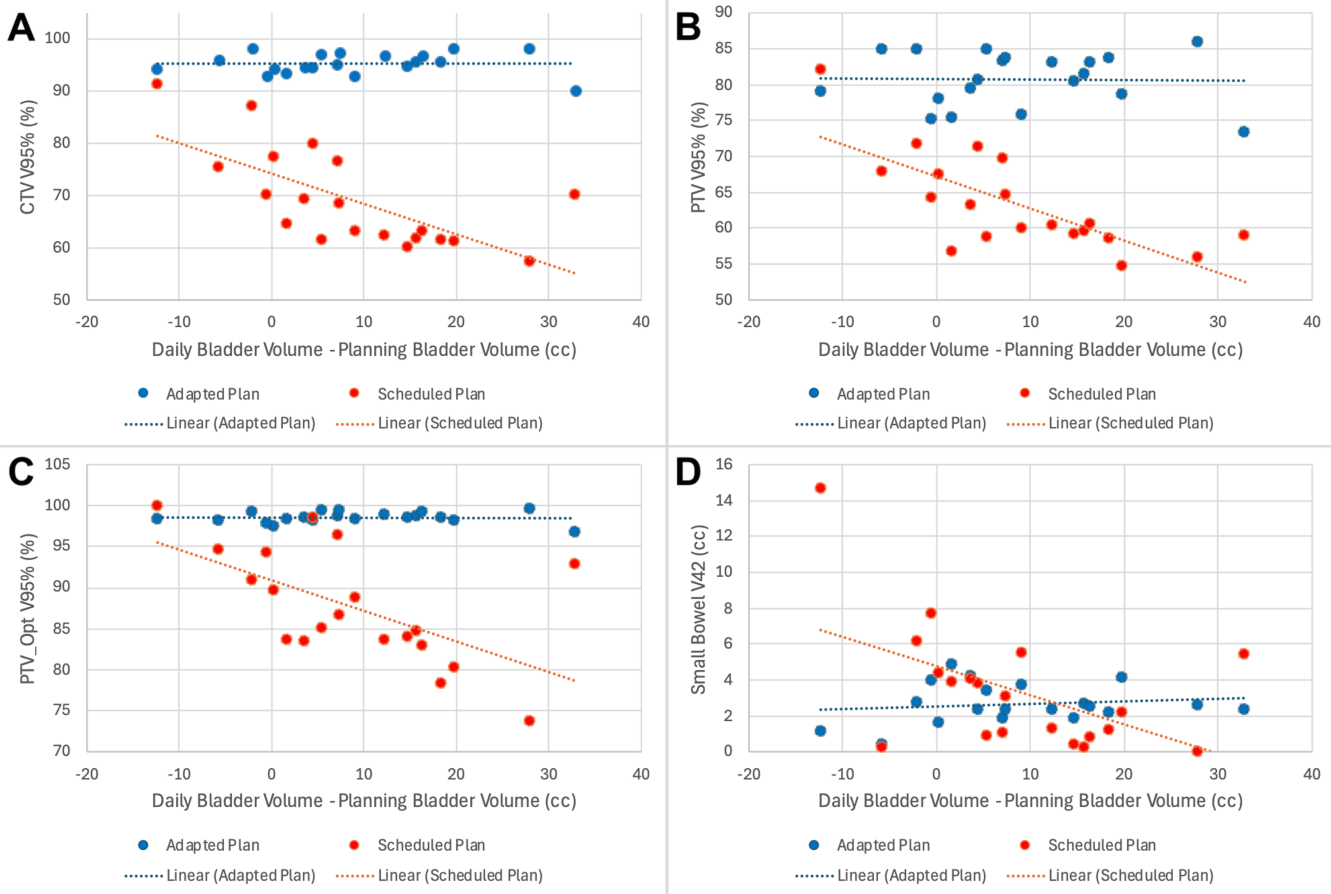
Daily dosimetry, as a function of the difference in volume of the bladder on planning and treatment scans, for Patient 1 Daily CTV V95% (A), PTV V95% (B), PTV_Opt V95% (C), and Small Bowel V42 (D) are plotted against the difference in the volume of the bladder on planning and daily treatment scans CTV: clinical target volume; PTV: planning target volume; Opt: optimized

A summary of dosimetry over the course of treatment is shown in Table 1. When accounting for daily anatomical variation with CBCT, the mean optimized PTV V95% without adaptation was found to be 76%. After daily adaptation, a mean V95% of 98.5% was achieved. Daily changes in CTV V95%, PTV V95%, PTV_Opt V95%, and small bowel V42 are shown in Figure 4, which demonstrates markedly improved target coverage and a slightly better small bowel dose with adaptation. The impact of daily fluctuations in bladder size on these metrics is shown in Figure 5, which shows a significant detriment to coverage when the bladder is filled beyond its recorded volume at the time of initial simulation, and this can be adequately corrected with adaptation.

**Table 1:** Summary of target and organ-at-risk dosimetry in reference, scheduled, and adapted plans for Patient 1 PTV: planning target volume; CTV: clinical target volume; Opt: optimized

Structure	Objective	Reference plan	Scheduled plan	Scheduled plan	Adapted plan
			Base plan (1.5 cm PTV)	Mean ± standard deviation (Min-Max)	Mean ± standard deviation (Min-Max)
CTV5500 V100% (%)	>98%	89.1	82.9	66 ± 8.7 (54-89.2)	93.1 ± 2.8 (85.7-97.4)
CTV5500 V95% (%)	>95%	91.3	100	69.1 ± 9.4 (57.2-91.2)	95.3 ± 2.1 (90-98.1)
CTV5500 Dmax (%)	<110%	122.7	106.7	120.7 ± 1.2 (118.9-123.1)	116.7 ± 26.4 (11.8-147.2)
PTV5500 V100% (%)	>95%	76.5	72.8	60.1 ± 6.6 (51.4-78.6)	78.5 ± 4 (70.3-84.4)
PTV5500 V95% (%)	>95%	78.6	100	63.3 ± 6.7 (54.8-82.1)	80.7 ± 3.8 (73.3-86)
PTV5500 Dmax (%)	<110%	122.7	101.1	121.5 ± 1.6 (119.4-126.5)	122.5 ± 8.9 (113-147.2)
PTVOpt V100% (%)	>95%	95.1	89.2	72.7 ± 6.9 (61.3-91.4)	96.2 ± 1.3 (93-98.1)
PTVOpt V95% (%)	>95%	97.2	100	76.2 ± 6.8 (65.4-93.9)	98.5 ± 0.7 (96.8-99.5)
CTVOpt V100% (%)	>95%	100	93.8	87.6 ± 7 (73.7-100)	100 ± 0 (99.9-100)
PTV-CTV D5% (%)	<115%	109.8	104.4	108.1 ± 1.1 (106.3-110.5)	108.9 ± 1.1 (107.3-111.2)
Small Bowel V42Gy (cc)	<5 cc	2.27	64	3.3 ± 3.5 (0-14.6)	2.6 ± 1.1 (0.4-4.9)

Currently, the patient has completed six-month surveillance CT imaging without evidence of recurrent disease. A three-month post-treatment cystoscopy has been performed with similar findings, although the assessment was complicated by post-treatment changes involving the bladder mucosa.

Patient 2 (node-positive)

**Figure 6:**
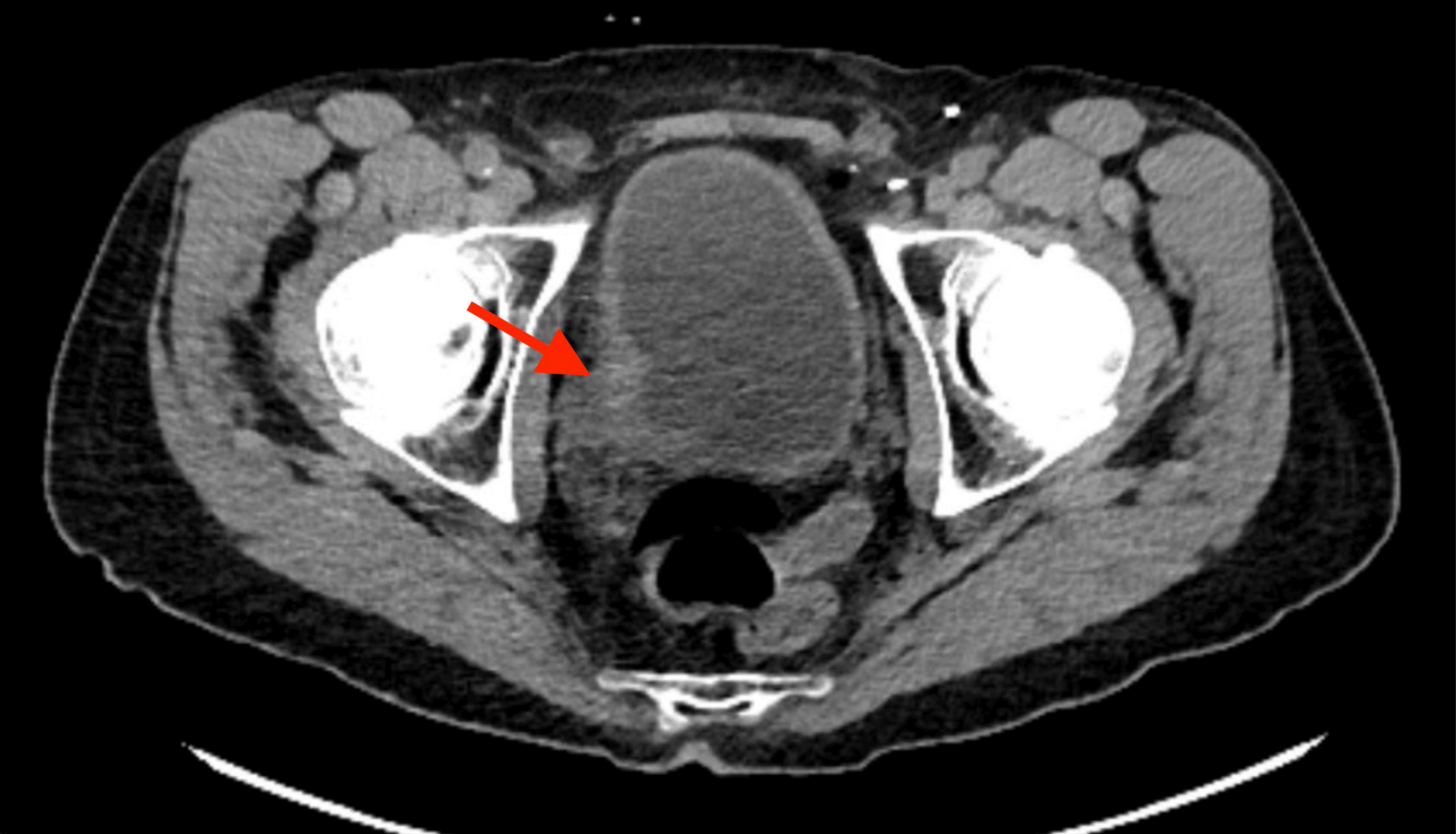
CT pelvis depicting enhancing mass within the right posterolateral wall of the bladder CT: computed tomography

A 78-year-old man with a past medical history including hypertension and chronic obstructive pulmonary disorder began workup in late 2023 after developing gross hematuria. Initial CT of the abdomen and pelvis revealed mass-like wall thickening of the right posterolateral urinary bladder (Figure 6). A CT urogram was subsequently obtained, which showed an enhancing mass within the right posterolateral wall of the bladder, with an exophytic component extending towards the right hemipelvis, nearly abutting the right obturator internus muscle. A subsequent cystoscopy confirmed findings concerning malignancy involving the right bladder wall.

**Figure 7:**
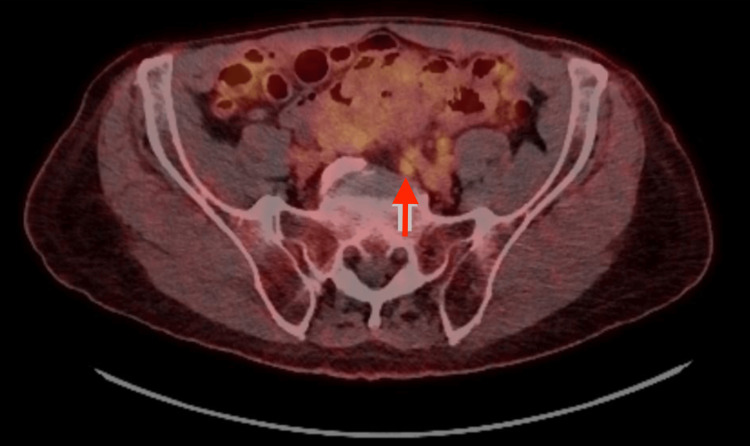
FDG-PET/CT depicting suspicious common iliac lymph node FDG: fludeoxyglucose F18; PET: positron emission tomography; CT: computed tomography

He later underwent TURBT, with pathology consistent with high-grade urothelial carcinoma with invasion through the muscularis propria, and a focus suspicious for lymphovascular invasion. A staging FDG-PET/CT showed findings consistent with known malignancy, as well as an avidly enlarged left-sided common iliac lymph node (Figure 7). The right renal pelvis and ureter were dilated and did not contain significant urinary activity, which was concerning for an obstructive process at the ureterovesical junction. He, therefore, underwent right nephrostomy tube placement given hydroureteronephrosis.

The patient met with medical oncology, who recommended upfront neoadjuvant systemic treatment. Given post-renal acute kidney injury secondary to ureterovesicular involvement, the patient was recommended to complete four cycles of enfortumab vedotin with pembrolizumab. After completing his fourth cycle, the patient completed a restaging PET/CT, which showed the resolution of a previously avid and enlarged left common iliac lymph node. There was minimal change in the appearance of the known bladder primary.

The patient met with urology to discuss further management, with treatment options including cystectomy, pelvic lymph node dissection, and urinary diversion. The patient expressed a preference for radiation at this time, with the intent to preserve his bladder and prioritize quality of life. A repeat cystoscopy was performed, and no tumor was identified. The patient was subsequently referred to radiation oncology to discuss non-operative management.

Out of concern for nodal involvement, the patient was offered concurrent chemoradiation. The pelvic lymph nodes would be treated to 45 Gy in 1.8 Gy per fraction, with a simultaneous integrated boost to the bladder and suspicious lymph nodes to 57.5 Gy in 2.3 Gy per fraction. Concurrent chemotherapy would include cisplatin/5-FU. The patient underwent a CT simulation with an empty bladder protocol. Similarly to the prior patient, the treating physician was responsible for generating the CTV and PTV treatment volumes, while the therapist contoured the OARs, which were later adjusted by the MD. The entire bladder CTV was delineated. The suspicious lymph node was contoured using the planning CT with the assistance of fused PET/CT data. The pelvic lymphatics CTV was contoured to include the common iliac, internal and external iliac, and presacral regions. OARs contoured included the small bowel, bilateral femoral heads, and rectum. For the reference plan, a 1.5 cm bladder PTV expansion was used, whereas a 0.7 cm expansion was used for the adaptive plan. In both plans, the node and pelvic lymphatics received a 0.7 cm PTV expansion.

**Figure 8:**
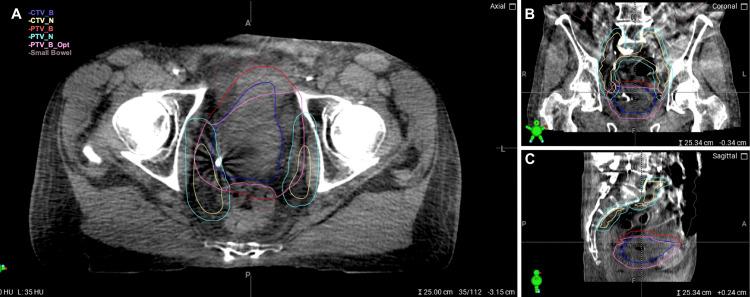
Contours for Patient 2 Axial (A), sagittal (B), and coronal (C) depictions of relevant contours CTV: clinical target volume; PTV: planning target volume; Opt: optimized

There was a similar concern for bowel toxicity in this patient. Given the pelvic lymph node involvement necessitating coverage of regional pelvic lymphatics, a considerable volume of bowel would receive a partial prescription radiation dose. Variation in bladder anatomy daily would confer a similar risk to the small bowel. To optimize bowel dose, the patient was offered treatment utilizing a CT-guided online adaptive radiotherapy technique, using the same type of approach/workflow described in the first case. Though minimal imaging artifact was present, it did not impact the ability to contour targets and OARs. Again, contouring took approximately 10-15 minutes per fraction and was performed on the daily CBCT. For the suspicious lymph node and bladder, it was ensured that CT correlates were encompassed in the CTVs. Target volumes are shown in Figure 8.

**Figure 9:**
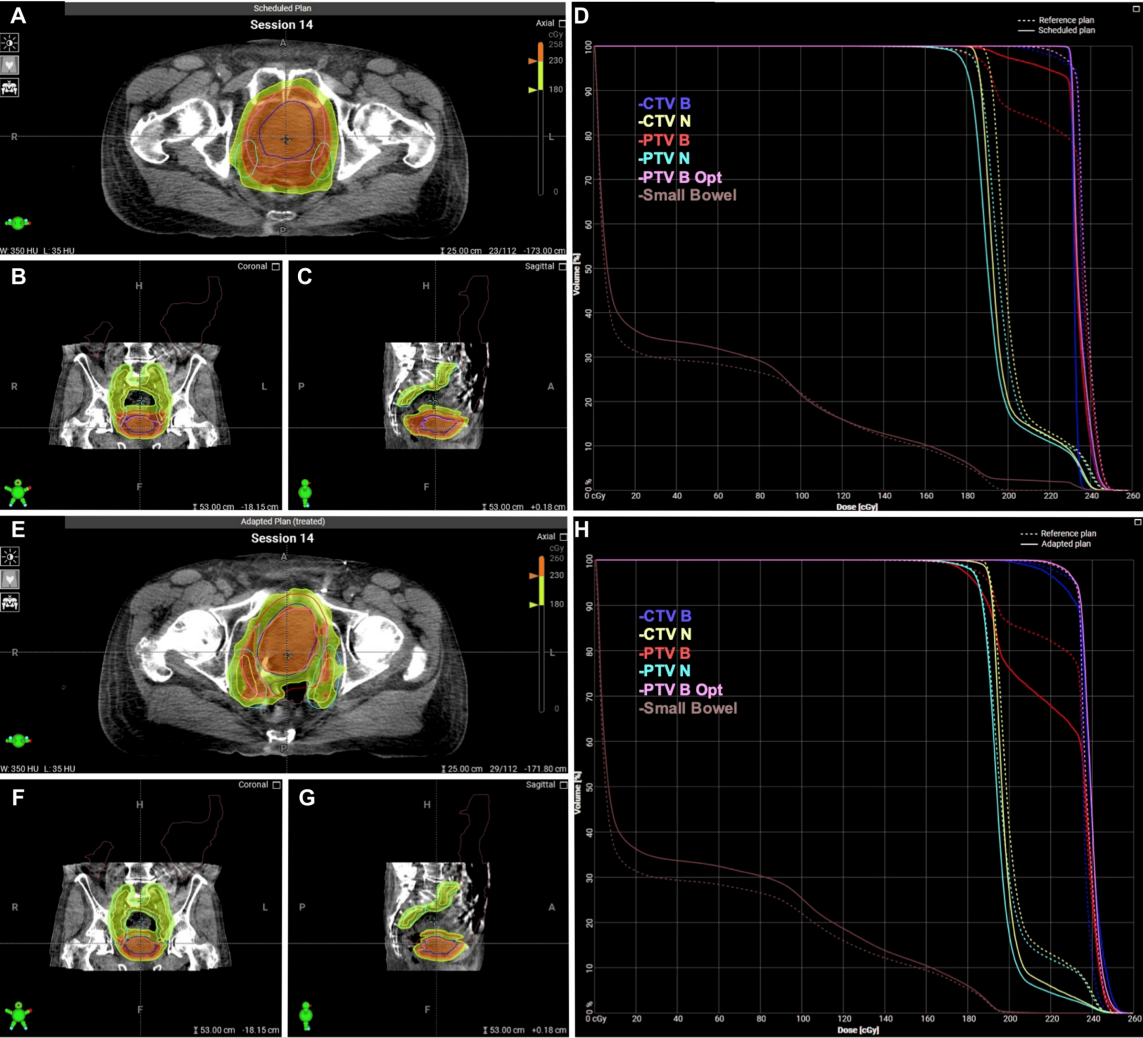
Scheduled and adapted radiation treatment plans for Patient 2 Axial (A), sagittal (B), and coronal (C) depictions of scheduled plan. Dose-volume histogram (D) of scheduled plan. Axial (E), sagittal (F), and coronal (G) depictions of the scheduled plan. Dose-volume histogram (H) of the adapted plan. CTV: clinical target volume; PTV: planning target volume; Opt: optimized

The patient completed his course without significant toxicity. He reported mild CTCAE grade 1 fatigue. The patient did have UTIs throughout his treatment course due to nephrostomy exchanges, which complicated the assessment of radiation cystitis. Due to a combination of target coverage and dose to the small bowel, the adapted plan was selected for all 25 fractions. The median patient time on the table was 36 minutes (range 24-51). Examples of the initial plan and adapted plan for a given fraction’s anatomy are shown in Figure 9.

**Figure 10:**
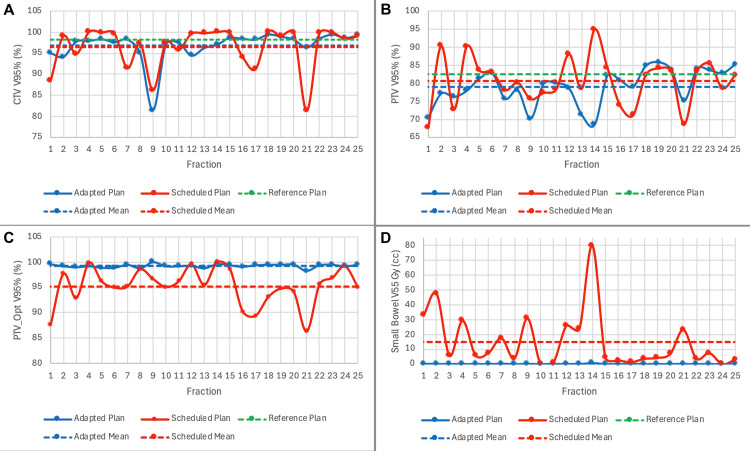
Daily dosimetry for target volumes and organs at risk for Patient 2 Daily CTV V95% (A), PTV V95% (B), PTV_Opt V95% (C), and Small Bowel V55 (D) are plotted over the course of treatment CTV: clinical target volume; PTV: planning target volume; Opt: optimized

**Figure 11:**
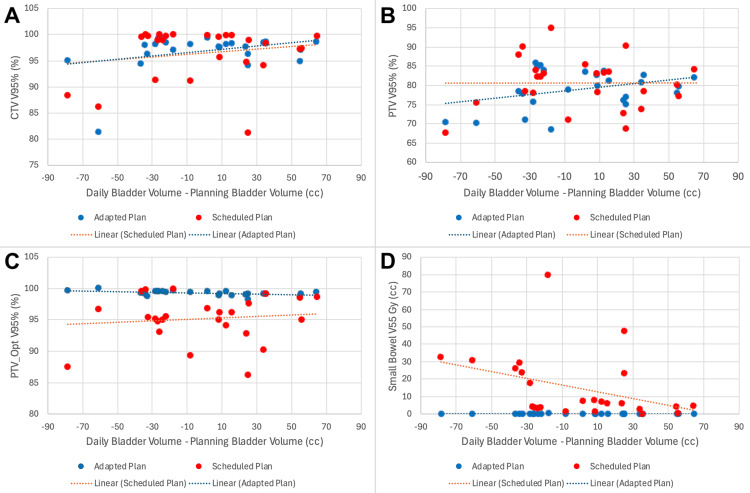
Daily dosimetry, as a function of the difference in volume of the bladder on planning and treatment scans, for Patient 2 Daily CTV V95% (A), PTV V95% (B), PTV_Opt V95% (C), and Small Bowel V55 are plotted against the difference in the volume of the bladder on planning and daily treatment scans CTV: clinical target volume; PTV: planning target volume; Opt: optimized

A summary of dosimetry over the course of treatment is shown in Table 2. When accounting for daily anatomical variation with CBCT, the mean optimized PTV V95% without adaptation was 95.2%. After daily adaptation, a mean V95% of 99.3% was achieved. Additionally, the dose delivered to the small bowel was significantly reduced. The scheduled plan would have delivered a V55 Gy of 14.9 cc. Daily adaptation was able to reduce the volume receiving 55 Gy or higher to 0.1 cc. Daily changes in CTV V95%, PTV V95%, PTV_Opt V95%, and small bowel V55 are shown in Figure 10, which demonstrates markedly improved target coverage and a slightly better small bowel dose with adaptation. The impact of daily fluctuations in bladder size on these metrics is shown in Figure 11, which illustrates the potential detriment to coverage when the bladder is filled beyond its recorded volume at the time of the initial simulation. Overfilling can be adequately corrected with adaptation.

**Table 2:** Summary of target and organ-at-risk dosimetry in reference, scheduled, and adapted plans for Patient 2 PTV: planning target volume; CTV: clinical target volume; Opt: optimized

Structure	Objective	Reference plan	Scheduled plan	Adapted plan
			Mean ± standard deviation (Min-Max)	Mean ± standard deviation (Min-Max)
PTV_Bladder_5750 V100% (%)	>95%	78.9	73.1 ± 9 (55.3-90.2)	75.2 ± 5.2 (63.3-82.1)
PTV_Bladder_5750 V95% (%)	>95%	82.5	80.6 ± 6.8 (67.7-94.9)	79 ± 5 (68.5-85.8)
PTV_Bladder_5750 Dmax (%)	<110%	112.3	390.5 ± 1399.9 (107.7-7109.9)	110.9 ± 2.8 (108.6-122.9)
PTV_Nodes_4500 V100% (%)	>95%	98.5	94.3 ± 3 (82.6-97.8)	98.2 ± 0.3 (97.1-98.8)
PTV_Nodes_4500 V95% (%)	>95%	99.5	98.5 ± 1.3 (92.8-99.5)	99.4 ± 0.4 (98-99.8)
CTV_Bladder_5750 V100% (%)	>95%	95.7	87.4 ± 15.3 (37.4-99.6)	93.8 ± 4.2 (78.2-98)
CTV_Bladder_5750 V95% (%)	>95%	98.2	96.4 ± 5 (86.2-100)	96.8 ± 3.6 (81.4-99.4)
CTV_Bladder_5750 Dmax (%)	<110%	109.1	108.5 ± 1.6 (105.1-111.5)	109.8 ± 1.8 (107.5-115.4)
CTV_Nodes_4500 V100% (%)	>95%	100	99.7 ± 0.9 (95.4-100)	99.9 ± 0.2 (99.3-100)
CTV_Nodes_4500 V95% (%)	>95%	100	100 ± 0.1 (99.5-100)	100 ± 0.1 (99.5-100)
PTV4500_Opt V100% (%)	>95%	98.3	90.2 ± 18.6 (2.2-97.6)	98.1 ± 0.3 (97-98.7)
PTV4500_Opt V95% (%)	>95%	99.4	98.3 ± 1.4 (92.5-99.4)	99.3 ± 0.4 (97.9-99.8)
PTV4500-CTV4500 V6300 (cc)	<0.02 cc	0	0 ± 0 (0-0)	0.1 ± 0.2 (0-0.7)
CTV4500_Opt V100% (%)	>95%	100	99.7 ± 1 (94.8-100)	99.9 ± 0.2 (99.2-100)
CTV5750_Opt V100% (%)	>95%	100	93.2 ± 16.3 (35.6-100)	100 ± 0 (100-100)
PTV5750_Opt V100% (%)	>95%	96.2	88 ± 9.4 (63.2-98.7)	96.1 ± 0.9 (94.7-99.5)
PTV5750_Opt V95% (%)	>95%	99.3	95.2 ± 3.7 (87.6-100)	99.3 ± 0.3 (98.8-100)
PTV5750_Opt-CTV5750_Opt Dmax (%)	<110%	112.3	110.5 ± 1.2 (107.7-112.7)	110.9 ± 2.8 (108.6-122.9)
Small Bowel V5500 (cc)	<0.02 cc	0	14.9 ± 18.7 (0.4-79.8)	0.1 ± 0.1 (0-0.7)
Small Bowel V5000 (cc)	<3 cc	3.42	25.4 ± 21.3 (4.3-95.5)	5.8 ± 2.4 (1.5-11.6)
Rectum V6050 (cc)	<0.02 cc	0.02	1 ± 2.2 (0-10.8)	0 ± 0.1 (0-0.3)
Rectum V6000 (cc)	<3 cc	0.13	1.8 ± 3.4 (0-17.1)	0.1 ± 0.2 (0-0.7)
Rectum Dmean (Gy)	<2500 Gy	97	123.1 ± 24.6 (88-204)	96 ± 14.2 (65-146)

The patient is currently scheduled to follow up with his first set of surveillance imaging.

## Discussion

The cases discussed demonstrate attempts to improve dosimetry to OARs without compromising target coverage and oncologic outcomes. This improved dosimetry would be expected to translate to reduced long-term OAR toxicity, although it has yet to be validated in a prospective fashion. Following curative treatments, patients subsequently focus on the side effects of treatment and their impact on quality of life. As previously discussed, approximately 20% of patients who undergo a bladder preservation approach deal with persistent bowel symptoms. The toxicity of the organ requiring treatment is expected to a degree but ideally should be minimized to the surrounding organs.

The therapeutic ratio, defined as the likelihood of tumor control in relation to normal tissue damage, continues to improve with technological advances. These presented cases highlight modern opportunities for improvement - especially when delivering radiation within the pelvic compartment. Currently, many methods are used to minimize the variability of internal anatomy to ensure fidelity of dose delivery, including fasting prior to CT simulation and treatment, respiratory management, full and empty bladder protocols, and defecation prior to receiving treatment. However, as demonstrated in these two cases, even when multiple of these methods are utilized, substantial internal anatomical variation can still occur.

With further improvements in the accuracy of treatment, the late side effect profile with radiation is expected to become more favorable. With the advent of adaptive radiation, dose escalation attempts within the bladder space are underway [17-19]. Other studies have also investigated the impact of this modality on toxicity outcomes [20].

These cases highlight multiple strengths of adaptive technology. First, the opportunity to use smaller PTV margin expansions. Previous PTV expansions historically were as large as 2 cm out of necessity. Current work is being done to assess PTV margin expansions as small as 5 mm [15]. This decreased expansion is feasible within the confines of an adaptive setting. This can be seen in the comparison of the scheduled plan in relation to the adapted plan in our first patient, in which a substantial decrease in coverage to the PTV would occur without adaptation. Secondly, both scheduled plans show marked variability in the location of pelvic contents, including both the target (bladder) and OARs. The dosimetric impact can be seen with and without daily adaptation (Figures 4, 5, 10, 11).

Adaptive radiotherapy in the context of bladder cancer treatment should accomplish equivalent CTV coverage while improving dosimetry to OARs through two primary means. The first is the substantial reduction in the necessary PTV expansion. To appropriately evaluate this, the reference non-adaptive plan using a 1.5 cm PTV expansion must be compared to the 0.5 cm PTV expansion of the adapted plan. The resultant reduction of dose to the OARs of concern can then be seen. Additional OAR dosimetric benefit manifests through daily adaptation, although it is not as readily appreciable when comparing the scheduled to the adapted plan, as both plans include the reduced PTV margin.

Finally, daily adaptive radiotherapy does introduce an increased workload that requires appropriate delegation and utilization of available resources. For more protracted courses longer than five fractions, we provided initial training and instruction to ancillary staff in contouring. For the initial fraction, the treating physician contoured the bladder and relevant OARs. For subsequent fractions, therapists and physicists completed preliminary contours, which were adjusted and/or verified by the physician. The treating physician then reviewed the generated plan prior to treatment delivery. Our experience is that adaptive radiotherapy is feasible for even fractionated bladder cancer cases (both re-irradiation and node-positive) from a resource utilization perspective.

## Conclusions

TMT for bladder cancer is an attractive management option with curative and organ-sparing intent. Traditional 3D conformal radiation delivery can result in long-term bowel toxicity in a subset of patients treated with this modality. Our case series demonstrates the feasibility of adaptive radiotherapy as treatment for MIBC, specifically in the re-irradiation setting (where reduction of dose to OARs is even more important) and in a node-positive patient (where more extensive target volumes are required and must be considered when aiming to reduce doses to OARs). Delivery of radiation with adaptive technology can improve the cumulative dosimetry of OARs without compromising the coverage of the target of interest. Though online-adaptive treatment represents a logistical challenge for fractionated courses of radiation, as is the case for bladder cancer, the workflows we present demonstrate the feasibility of the adaptive approach in such patients.

## References

[1] Chang SS, Bochner BH, Chou R (2017). Treatment of non-metastatic muscle-invasive bladder cancer: AUA/ASCO/ASTRO/SUO guideline. J Urol.

[2] Bochner BH, Dalbagni G, Marzouk KH (2018). Randomized trial comparing open radical cystectomy and robot-assisted laparoscopic radical cystectomy: oncologic outcomes. Eur Urol.

[3] Rai BP, Bondad J, Vasdev N (2019). Robotic versus open radical cystectomy for bladder cancer in adults. Cochrane Database Syst Rev.

[4] Parekh DJ, Reis IM, Castle EP (2018). Robot-assisted radical cystectomy versus open radical cystectomy in patients with bladder cancer (RAZOR): an open-label, randomised, phase 3, non-inferiority trial. Lancet.

[5] Patel VG, Oh WK, Galsky MD (2020). Treatment of muscle-invasive and advanced bladder cancer in 2020. CA Cancer J Clin.

[6] Zlotta AR, Ballas LK, Niemierko A (2023). Radical cystectomy versus trimodality therapy for muscle-invasive bladder cancer: a multi-institutional propensity score matched and weighted analysis. Lancet Oncol.

[7] Mak RH, Hunt D, Shipley WU (2014). Long-term outcomes in patients with muscle-invasive bladder cancer after selective bladder-preserving combined-modality therapy: a pooled analysis of Radiation Therapy Oncology Group protocols 8802, 8903, 9506, 9706, 9906, and 0233. J Clin Oncol.

[8] Choudhury A, Porta N, Hall E (2021). Hypofractionated radiotherapy in locally advanced bladder cancer: an individual patient data meta-analysis of the BC2001 and BCON trials. Lancet Oncol.

[9] Zietman AL, Sacco D, Skowronski U (2003). Organ conservation in invasive bladder cancer by transurethral resection, chemotherapy and radiation: results of a urodynamic and quality of life study on long-term survivors. J Urol.

[10] Mak KS, Smith AB, Eidelman A (2016). Quality of life in long-term survivors of muscle-invasive bladder cancer. Int J Radiat Oncol Biol Phys.

[11] McDonald F, Waters R, Gulliford S, Hall E, James N, Huddart RA (2015). Defining bowel dose volume constraints for bladder radiotherapy treatment planning. Clin Oncol (R Coll Radiol).

[12] Foroudi F, Wong J, Kron T (2011). Online adaptive radiotherapy for muscle-invasive bladder cancer: results of a pilot study. Int J Radiat Oncol Biol Phys.

[13] Tuomikoski L, Collan J, Keyriläinen J, Visapää H, Saarilahti K, Tenhunen M (2011). Adaptive radiotherapy in muscle invasive urinary bladder cancer--an effective method to reduce the irradiated bowel volume. Radiother Oncol.

[14] de Mol van Otterloo SR, Christodouleas JP, Blezer EL (2020). The MOMENTUM study: an international registry for the evidence-based introduction of MR-guided adaptive therapy. Front Oncol.

[15] Hafeez S, Webster A, Hansen VN (2020). Protocol for tumour-focused dose-escalated adaptive radiotherapy for the radical treatment of bladder cancer in a multicentre phase II randomised controlled trial (RAIDER): radiotherapy planning and delivery guidance. BMJ Open.

[16] James ND, Hussain SA, Hall E (2012). Radiotherapy with or without chemotherapy in muscle-invasive bladder cancer. N Engl J Med.

[17] Murthy V, Gupta P, Baruah K (2019). Adaptive radiotherapy for carcinoma of the urinary bladder: long-term outcomes with dose escalation. Clin Oncol (R Coll Radiol).

[18] Murthy V, Masodkar R, Kalyani N (2016). Clinical outcomes with dose-escalated adaptive radiation therapy for urinary bladder cancer: a prospective study. Int J Radiat Oncol Biol Phys.

[19] Huddart R, McDonald F, Hafeez S (2014). Phase I dose-escalated image-guided adaptive bladder radiotherapy study: results of first dose cohort (68Gy). J Clin Oncol.

[20] Yeh J, Bressel M, Tai KH, Kron T, Foroudi F (2021). A retrospective review of the long-term outcomes of online adaptive radiation therapy and conventional radiation therapy for muscle invasive bladder cancer. Clin Transl Radiat Oncol.

